# How does cognitive function measured by the reaction time and critical flicker fusion frequency correlate with the academic performance of students?

**DOI:** 10.1186/s12909-020-02416-7

**Published:** 2020-12-14

**Authors:** Archana Prabu Kumar, Abirami Omprakash, Maheshkumar Kuppusamy, Maruthy K.N., Sathiyasekaran B.W.C., Vijayaraghavan P.V., Padmavathi Ramaswamy

**Affiliations:** 1 Present Address: Medical Education Unit, College of Medicine and Medical Sciences, Arabian Gulf University, Manama, Bahrain; 2 Department of Physiology, Sri Ramachandra Medical College and Research Institute, Sri Ramachandra Institute of Higher Education and Research, Porur, Chennai, Tamil Nadu India; 3 Department of Biochemistry and Physiology, Government Yoga and Naturopathy Medical College and Hospital, Chennai, Tamil Nadu India; 4 Department of Physiology, Narayana Medical College, Nellore, India; 5 Department of Community Medicine, Sri Ramachandra Medical College and Research Institute, Sri Ramachandra Institute of Higher Education and Research, Porur, Chennai, Tamil Nadu India; 6 Department of Orthopaedics, Sri Ramachandra Medical College and Research Institute, Sri Ramachandra Institute of Higher Education and Research, Porur, Chennai, Tamil Nadu India

**Keywords:** Auditory reaction time (ART), Visual reaction time (VRT), Critical flicker fusion frequency (CFFF), Academic performance

## Abstract

**Background:**

The reaction time (RT) is “*the time taken for the appearance of rapid voluntary reaction by an individual following a stimulus, either auditory or visual*” and the Critical Flickering Fusion Frequency (CFFF) is “*the rate at which successively presented light stimuli appear to be steady and continuous*”. RT and CFFF are commonly used for the assessment of cognitive functions that are known to influence academic performance. However, data about the exact correlation between these are scarce, particularly in India. This research aimed to study the association between visual RT (VRT), auditory RT (ART) and CFFF and their impact on the academic performance of undergraduate students.

**Methods:**

This cross-sectional study was conducted on 700 students of Faculty of Medicine and Dentistry at a private medical university in South India, during the period from 2015 to 2017. The VRT, ART and CFFF were evaluated, and the best out of three subsequent attempts was recorded. The mean score (in percentage) of the three best marks out of the five internal assessments for the course during each academic year was considered for analysis. The association between the different cognitive tests and the average academic performance was analysed.

**Results:**

Female students had faster VRT (*n *= 345, mean = 243.97, SD = 83.87) than male students (*n* = 273, mean = 274.86, SD = 96.97) (*p* = 0.001). VRT and ART had a moderate negative correlation with academic performance (for ART, *r =* − 0.42, *p* < 0.001; for VRT; *r =* − 0.40, *p <* 0.001). CFFF had a very weak positive correlation with academic performance (*r =* 0.19, *p* = 0.01). The only independent predictors of academic performance were RT and gender (Adjusted R^2^ = 0.11).

**Conclusion:**

Although there is a correlation between CFFF and cognitive function, our study showed only a weak correlation between CFFF and academic performance. Female students had faster RTs, and gender was an independent predictor of academic performance. Rather, students with faster RTs appear to have an advantage in academic performance.

## Background

Several factors can influence the academic performance of students, and cognitive ability is among the most important of these factors [1, 2]. Cognitive ability is determined by cognitive functions, which are in turn influenced by the speed of information processing, attention span, language skills, visual-spatial orientation and so on [3]. Many factors that influence cognitive functions have been identified. Some of the important factors include age, gender, body mass index (BMI), educational background, socio-economic status, hormonal disorders and other comorbid conditions [4]. In addition, it has been established that the aforementioned factors also impact academic performance [5].

The assessment of cognitive functions may help in the evaluation of students’ functional capacity [6]. There are many tools for the assessment of cognitive functions, out of which Reaction Time (RT) and Critical Flicker Fusion Frequency (CFFF) are two of the commonly used tests for the assessment of certain cognitive functions that are involved in learning and performance [7, 8]. RT and CFFF have also been documented as markers of a higher cognitive function [7–11]. They are commonly used in clinical settings because they are simple, reliable, valid and cheap [8, 12].

RT is the time elapsed between the presentation of a particular sensory stimulus to an individual and their consequent behavioural response to that stimulus [7]. This stimulus may be visual, auditory, tactile or that of any sensory modality [7]. The human body responds to different stimuli at various speeds [12]. For instance, it responds faster to auditory stimuli than to visual stimuli [12]. RT is a fundamental contributor to the processing of information, and it is considered to be an index for information processing speed and the speed of response programming [13]. Several types of RTs exist, such as the simple RT (time between the stimulus presentation and an individual’s response), recognition RT (response to a particular stimulus and not to others) and choice RT (different stimuli requiring different responses) [14].

The CFFF test is another tool for the assessment of cognitive domain. It evaluates the cortical arousal state and the activity of the central nervous system [8]. The visual cortex processes the sensory information it receives in two manners: temporal and spatial [15]. Spatial processing refers to the ability of the cortex to discriminate between different presenting stimuli with regard to their location in space, whereas temporal processing relates to the discrimination between the stimuli with respect to the time elapsed between them [15]. The CFFF test evaluates the visual cortex’s temporal processing of the stimuli. It measures the frequency of presentation of successive visual stimuli at which they are perceived as a continuous and stable stimulation rather than discrete events. Several studies show that CFFF is positively correlated with concentration, alertness, enhanced attention and executive functions [11, 16, 17]. Thus, it is used as an adjunct test for the evaluation of various cognitive domains during psychometric tests [11]. The CFFF test also has the advantages of being simple, easy to perform and language-independent [8].

Learning is a complex process that depends on several factors, namely concentration, arousal of the cerebral cortex, attentiveness and rapidity of information processing [18]. The sequence of events contributing to the measurement of RT includes the perception of the sensory stimulus, processing of the information through central and peripheral mechanisms and the passage of the motor impulse through the neuronal pathways, followed by motor activity (end-organ activation) [19]. RT evaluates the pace and quality of information processing [20], whereas CFFF measures the “cortical arousal” [21–23]. All of these play a vital role in effective learning, thereby facilitating better academic achievement.

Given the fact that the RT testing and the CFFF evaluation can measure information processing speed, attention, concentration and alertness [11, 13, 16, 17], all of which are important factors presumed to be associated with higher academic achievement [24–26], it can be expected that better cognitive functions may also be linked with better academic performance [27]. There are very few studies that have explored this relationship, and although they have shown that there is a statistically significant relationship between faster RT and better academic performance, the correlation appears to be weak at best (*r =* 0.07 to *r =* 0.29) [20, 28]. To the best of our knowledge, no studies have included CFFF. Therefore, this research aimed to study the association between the academic performance of undergraduate students in India and their visual RT (VRT), auditory RT (ART) as well as CFFF.

## Methods

This was a cross-sectional study conducted on undergraduate students who attended the Physiology and Applied Physiology course at the Faculty of Medicine and the Faculty of Dentistry at a private medical university in South India during the period from 2015 to 2017. Students with any visual problems, hearing deficiency, hormonal disorders or any neurological disease were excluded from the study. Ethical approval to carry out this research was obtained from the ethical committee of the host institution. After explaining the study details, written informed consent was taken from all the students who agreed to participate in the study.

Demographic data, such as age, gender and BMI, were collected from the students. The average academic score of the three best formal tests conducted during the academic year was calculated as an indication of academic performance. Auditory RT, visual RT and CFFF were then measured for each student.

### Academic performance

Five internal assessment exams relating to the discipline of Physiology and Applied Physiology were conducted at an interval of 8 to 10 weeks during each academic year. Each internal assessment included 15 multiple-choice questions (one mark each and a maximum total score of 15), four short notes (five marks each, maximum score of 20) and one essay (a maximum score of 15 marks). The maximum total score per internal assessment was 50. All the assessments were held for 1 h and 30 min each. All question papers were designed based on a standard blueprint, and the difficulty index was comparable for all the assessments. For this study, the mean score (in percentage) of the three best marks out of the five internal assessments was considered for analysis. No internal assessments were conducted on the same day as the cognitive tests. The tests were corrected by faculty members who were not aware of the results of the cognitive tests. The faculty members who corrected the answer scripts of the students were masked about the student’s RT and CFFF.

### Cognitive testing

The VRT and ART were assessed by using the PC 1000 Hz reaction timer, which is an in-house built device that comprises a 1000 Hz square wave oscillator [19]. The device is composed of a small light-emitting diode for visual stimulation, a headphone (1000 Hz) for auditory stimulation and two connected components (A and B), all of which are connected to a computer device. Component A is the part of the device that is controlled by the examiner via a start button, whereas component B is the part of the device that the subject faces. The RT was recorded via the Audacity software (version 1.2.2) in a 0.001 s accuracy wave format [29]. Our previous validation study on healthy volunteers using the PC 1000 Hz reaction timer showed a strong concurrent validity [19].

Prior to the recording, all subjects were instructed to get adequate sleep the night before the testing and have a light breakfast on the day of the test because sleep deprivation and the type of breakfast can affect cognition [30–32]. The students were also instructed not to consume any stimulants (including caffeinated foods and drinks) on the day of testing [33]. All recordings were done between 9 a.m. and 11 a.m. at the Physiology Department of the host institution, and all the students were educated about the tests prior to the testing.

#### Visual reaction time (VRT) testing

The VRT was measured in milliseconds (msec) by getting the subject to sit and look at component B of the PC 1000 Hz timer device and having the examiner sit in front of and control component A. The examiner used the start button on component A to start the stimulation procedure, and the subject was instructed to press the stop button with their dominant hand as soon as they saw the red light.

#### Auditory reaction time (ART) measurement

**Fig. 1:**
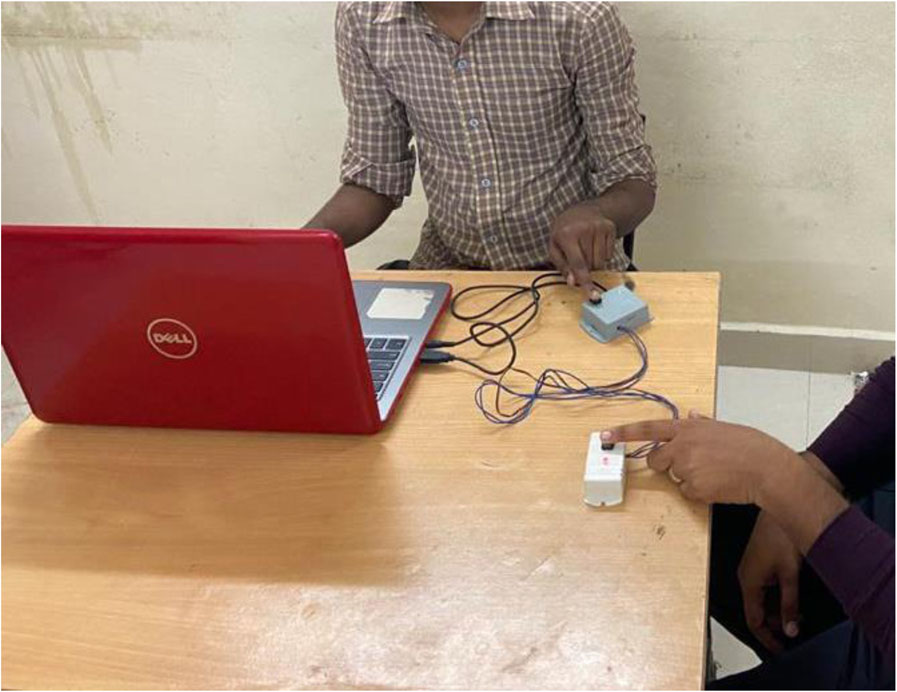
The procedure of testing the auditory reaction time. PC 1000 Hz reaction timer with component A (with examiner) and component B (with subject)

**Fig. 2:**
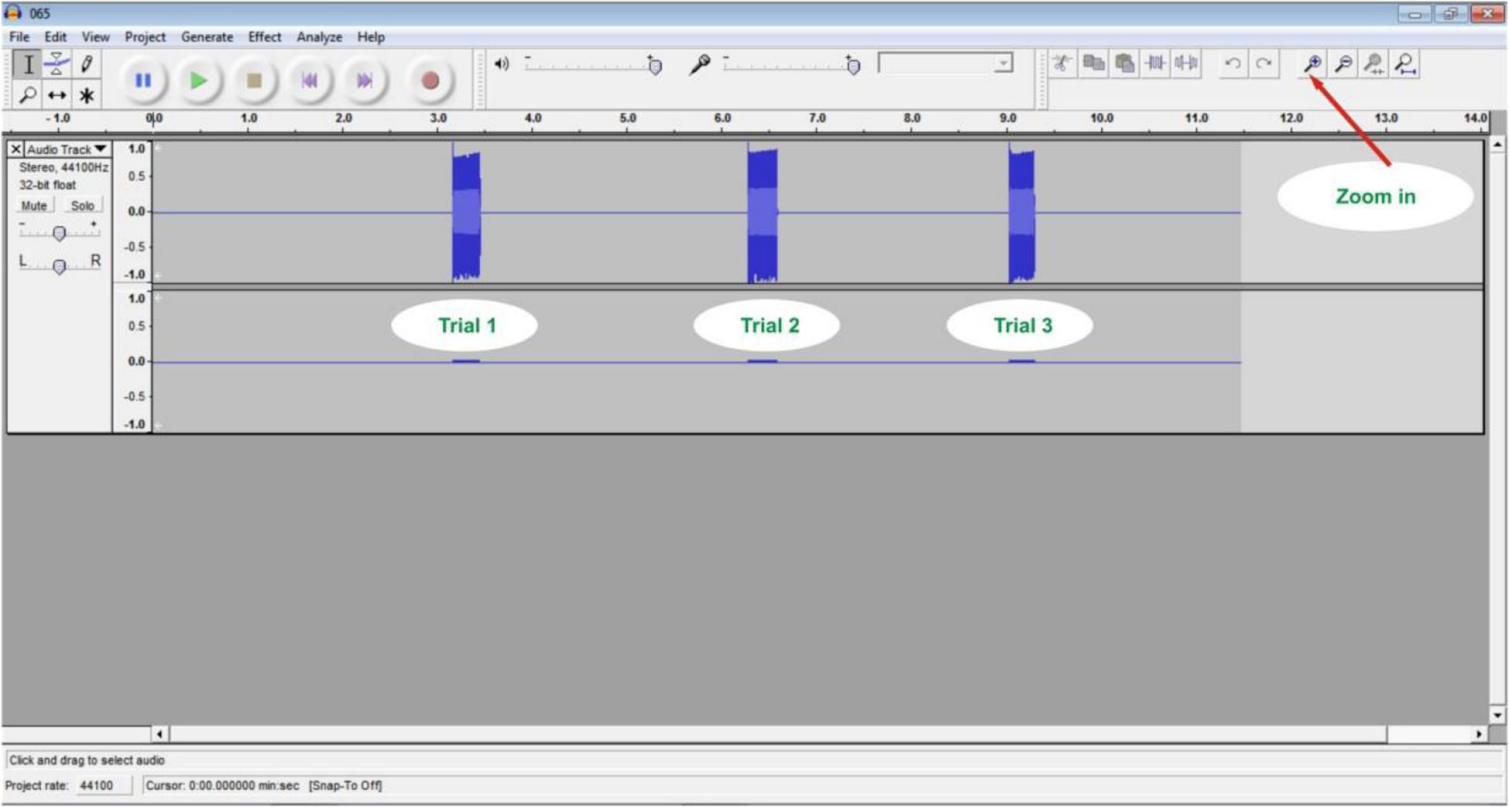
The audacity software used during ART and VRT measurement. Audacity software (version 1.2.2) storing the recordings of ART and VRT in a 0.001 s accuracy wave format

Similar to the case of the VRT measurement, the examiner started the stimulation by pressing the start button on component A of the device, and the subject pressed the stop button with their dominant hand once they heard the sound through their headphones (refer to Fig. 1). For each subject, three trials were allowed with an interval of one minute for both VRT and ART, and the minimum time recorded was the one used for analysis. The time elapsed between the presentation of the stimulus and the subject’s response was calculated in msec by using the Audacity software installed on the connected computer (refer to Fig. 2).

#### CFFF testing

**Fig. 3:**
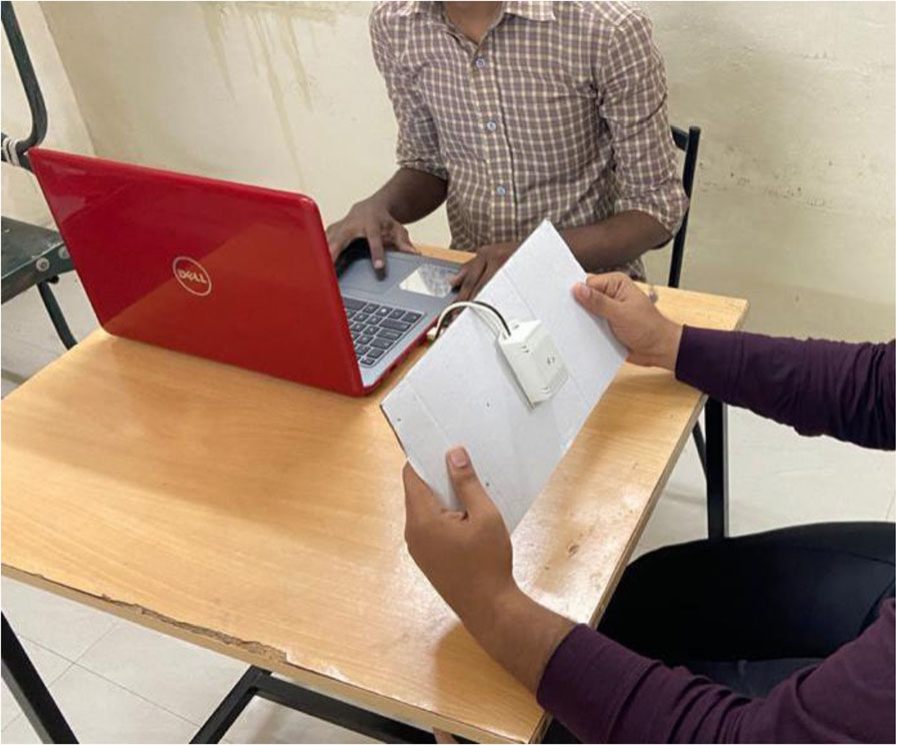
The procedure of CFFF measuring. CFFT test with red light against a white background with the subject sitting 80 cm away from the module

The CFFF test was carried out by using a standard electronic module and standard protocols as documented in other studies [21–23]. The system of this module presented a series of red-light stimuli at different frequencies ranging from 12 Hz to 120 Hz. The examination was conducted in a dimly lit room with the subject sitting 80 cm away from the module and a 40 W bulb fixed behind the subject. The red light was presented against a white background, and the frequency of the flicker was gradually increased from 12 Hz until the subject reported that the presented light was perceived as “steady”, “constant” or “fused” light (refer to Fig. 3). The mean value of three descending measures from high to low frequency when the subject reported that the light started to flicker and the mean value of three ascending measures from low to high frequency when the subject reported that the light stopped to flicker were collected for analysis.

### Statistical analysis

All the data were fed into the computer and analysed by using the Statistical Package for Social Science (SPSS) software, version 25.0 (IBM, Armonk, NY, USA). Data cleaning with the removal of outliers (RTs shorter than 95 msec or longer than 650 msec, academic scores of 0%) was performed prior to the analysis. Based on their average academic scores (“academic performance”), students were categorized as low achievers (i.e., they scored 35% or less), mid achievers (i.e., they scored between 36 and 74%) and high achievers (i.e., they scored higher than 75%). Based on their BMI, they were classified as underweight (BMI below 18.5), normal weight (BMI between 18.5 and 24.9) and overweight (BMI of 25 and above). The categorical variables (gender, academic performance group and BMI range) were presented as frequencies and percentages. The continuous variables (age, BMI, academic performance, ART, VRT and CFFF) were presented as means and standard deviations (SD) of the sample or medians and interquartile ranges (IQR). The academic performance was specified as the dependent variable. All other variables were considered as independent variables or factors.

Student’s t-tests were conducted for comparing the cognitive test results (ART, VRT and CFF) and the academic performance between the female and male students. One-way ANOVAs, followed by Tukey’s honestly significant difference (HSD) post hoc tests, were conducted to compare the ART and VRT of low, mid and high achievers as well as that of underweight, normal weight and overweight students. Pearson’s r correlation analysis was used to check for correlation between the academic results and ART, VRT and CFFF. The following convention was adopted to describe the strength of the correlation according to r values: 0.00 to 0.19 signifying “very weak”; 0.20 to 0.39 signifying “weak”; 0.40 to 0.59 signifying “moderate”; 0.60 to 0.79 signifying “strong”; 0.80 to 1.0 signifying “very strong”. Multiple regression analysis was conducted to examine the relative effects of CFFF, VRT, ART, BMI, and gender on academic performance. The statistical analysis was performed at a 0.05 level of significance. Complete case analysis was performed while dealing with missing data.

## Results

### Demographic characteristics and cognitive results

Seven hundred undergraduate students were recruited for this study. After data cleaning, 618 records were available for analysis. Female students constituted most of the recruited subjects (345 / 618, i.e., 55.8%). All students were 18-year-old (618 / 618), and hence, age was not considered further in any statistical test. Table 1 shows the ART, VRT, CFFF, BMI and academic performance of the study participants.

**Table 1:** Participants’ cognitive test results, BMI and academic performance (N-618)

Parameter	Median	Mean	1st Quartile	3rd Quartile
VRT (msec)	235.0	256.8	191.5	299.0
ART (msec)	215.0	235.1	169.0	285.0
CFFF (Hz)	26.00	26.69	23.00	30.00
BMI	23.15	23.72	20.37	26.37
Academic performance (%)	52.00	51.45	42.00	60.00

*n* number of participants, *BMI* body mass index, *VRT* visual reaction time, *ART* auditory reaction time, *CFFF* Critical Flicker Fusion Frequency

**Fig. 4:**
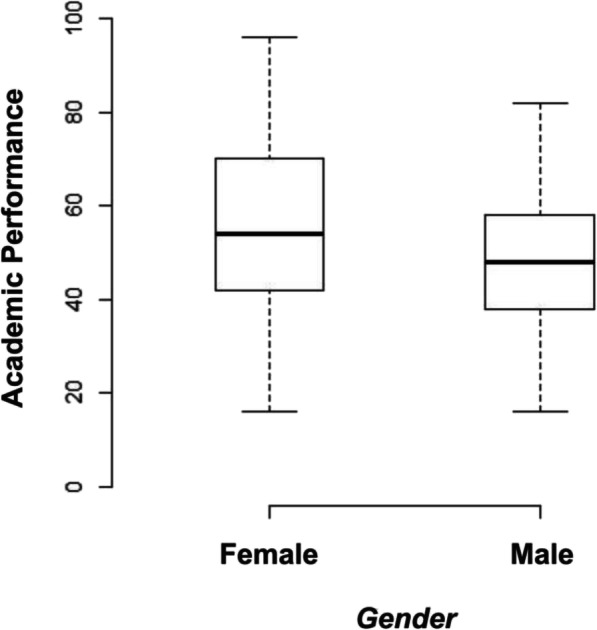
Boxplot of academic scores for female and male students. Female students had higher scores than male students, though the spread was more pronounced

Female students (*n* = 345) had a faster VRT (mean = 243.97, SD = 83.87) than male students (*n* = 273, mean = 274.86, SD = 96.97) (*p = 0.001*), and they demonstrated better academic performance (mean = 56.16, SD = 19.66) when compared to male students (mean = 48.02, SD = 13.13) (*p < 0.001*) (refer to Fig. 4). Although female students exhibited lower ART and CFFF measurements than male students, these did not reach statistical significance (refer to Table 2).

**Table 2:** Comparison between genders

Parameter	Female (*n* = 345)	Male (*n* = 273)	*P* value
VRT (msec)	243.97 ± 83.87	274.86 ± 96.97	0.001
ART (msec)	229.74 ± 76.84	242.01 ± 90.58	0.08
CFFF (Hz)	26.33 ± 5.16	26.93 ± 5.62	0.14
Academic performance (%)	56.16 ± 19.66	48.02 ± 13.13	0.0001

*Results in mean ± standard deviation, n* number of participants, *VRT* visual reaction time, *ART* auditory reaction time, *CFFF* Critical Flicker Fusion Frequency

A one-way ANOVA was performed to identify the effect of RT on exam performance for low, mid and high achievers (refer to Table 3). There was a significant effect of VRT [F (2, 615) = 6.40, *p =* 0.001] and ART [F (2, 615) = 24.47, *p =* 0.001] on exam performance. Tukey’s post hoc assessments showed that the RT of low achievers was significantly (*p* < 0.01) higher than the mid and high achievers. No such difference was found between the mid and high achievers (*p* > 0.05). There was also a statistically significant difference in the RTs between female and male students for each performance group (refer to Table 4).

**Table 3:** Comparison of academic performance groups

RT Parameter	Academic performance			Analysis of Variance between groups **		Pairwise comparisons (post hoc analysis ***) *P* value
	Low achievers (*N* = 101)	Mid achievers (*N* = 415)	High achievers (*N* = 102)	F	*P* value	
VRT (msec)	285.75 ± 91.7	260.36 ± 88.36	241.10 ± 69.13	6.40	0.001	Low–Mid = 0.01 Low–High = 0.001 Mid–High = 0.15
ART (msec)	278.75 ± 94.6	226.58 ± 74.8	208.5 ± 72.51	23.47	0.001	Low–Mid = 0.001 Low–High = 0.001 Mid–High = 0.13
CFFF (Hz)	25.68 ± 4.43	26.73 ± 5.54	31.27 ± 7.57	5.17	0.005	Low–Mid = 0.25 Low–High = 0.004 Mid–High = 0.01

p < 0.05, **analysis of variance, ***Tukey’s test

**Table 4:** Comparison between genders across different performance groups

Parameter	Female (*n =* 345)	Male (*n =* 273)	*P* value
VRT (msec)			
Low achievers	270.23 ± 89.35	300.31 ± 86.89	0.01
Mid achievers	246.35 ± 85.15	273.29 ± 89.80	0.02
High achievers	235.47 ± 65.44	256.67 ± 80.05	0.001
ART (msec)			
Low achievers	257.8 ± 95.62	298.92 ± 95.96	0.04
Mid achievers	225.05 ± 70.73	228.72 ± 78.55	0.01
High achievers	177.33 ± 53.28	212.39 ± 65.44	0.02

There was a statistically significant effect of VRT [F (2, 615) = 4.39, *p* = 0.01] on BMI, whereas no such effects were found in the case of ART [F (2, 615) = 0.02, *p* = 0.97]. Tukey’s post hoc assessments showed that students with normal weight have faster VRT, compared to underweight students (*p* < 0.05) but not compared to obese students (refer to Table 5).

**Table 5:** Comparison between BMI groups

RT Parameter	BMI			Analysis of Variance between groups **		Pairwise comparisons (post hoc analysis ***) *P* value
	Underweight (*n* = 69)	Normal (*n* = 340)	Obese (*n* = 209)	F	*P* value	
VRT (msec)	284.70 ± 96.45	249.92 ± 83.32*	257.39 ± 98.80	4.39	0.01	Normal–Underweight = 0.009 Normal–Obese–High = 0.51 Underweight–Obese = 0.09
ART (msec)	232.95 ± 72.98	235.41 ± 84.97	239.83 ± 82.15	0.02	0.97	Normal–Underweight = 0.97 Normal–Obese–High = 0.99 Underweight–Obese = 0.97
CFFF (Hz)	26.15 ± 5.32	26.77 ± 5.58	26.72 ± 5.42	0.39	0.67	Normal–Underweight = 0.65 Normal–Obese–High = 0.72 Underweight–Obese = 0.99

p < 0.05, **analysis of variance, ***Tukey’s test

*BMI* body mass index

### Correlations

**Fig. 5:**
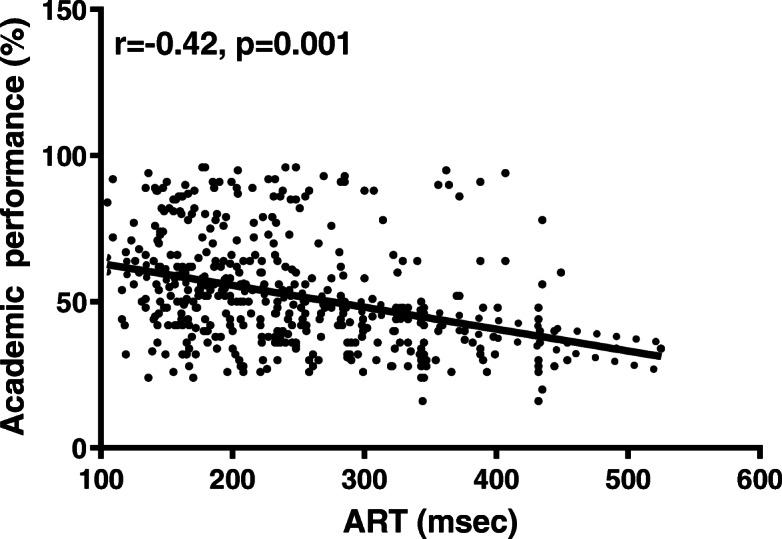
Scatter plot for ART and academic performance. Auditory Reaction Time (ART) had a moderate negative correlation with academic performance

**Fig. 6:**
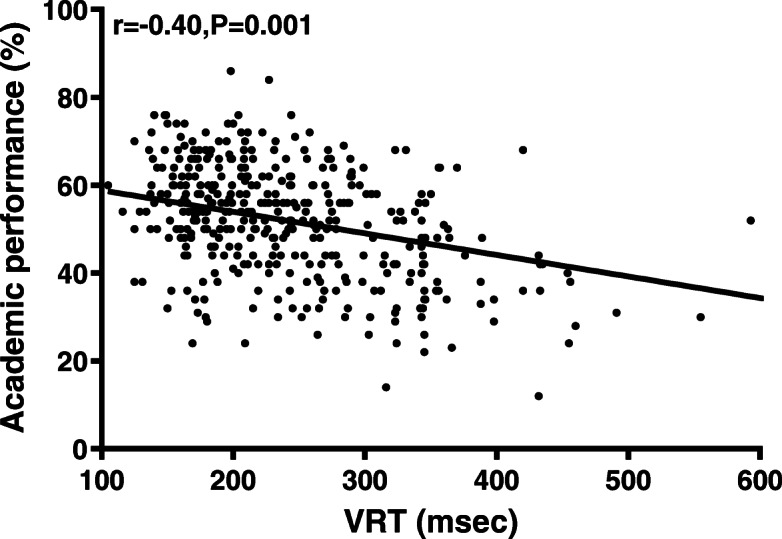
Scatter plot for VRT and academic performance. Visual Reaction Time (VRT) had a moderate negative correlation with academic performance

**Fig. 7:**
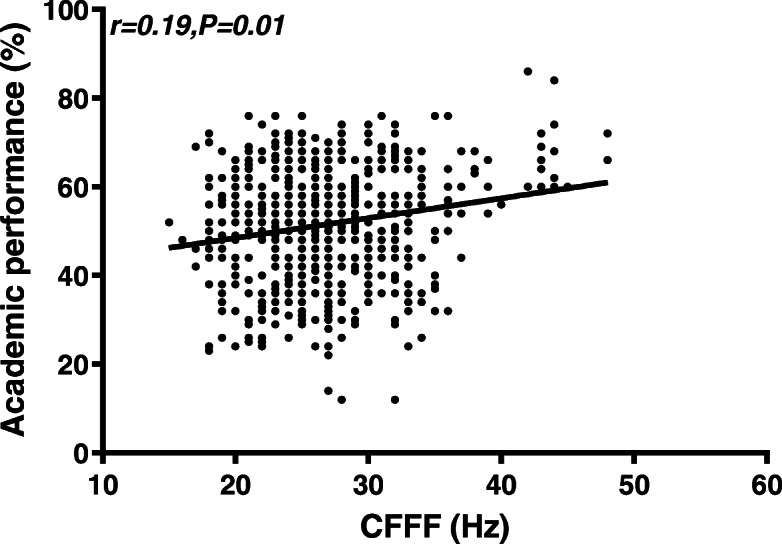
Scatter plot for CFFF and Academic performance. CFFF had a week positive correlation with academic performance

Pearson’s coefficient (r) was utilized to examine the correlation between academic performance and cognitive measurements (VRT, ART, CFFF). VRT and ART had a moderate negative correlation with academic performance (for ART, *r =* − 0.42, *p* < 0.001; for VRT; *r =* − 0.40, *p <* 0.001) (refer to Figs. 5 and 6). CFFF had a very weak positive correlation with academic performance (*r =* 0.19, *p =* 0.01; refer to Fig. 7). The correlations were quite similar while examining each gender separately (refer to Table 6).

**Table 6:** Correlation of academic performance with cognitive measurements for each gender

Parameter	Male		Female	
	R	*P* value	R	*P* value
VRT	−0.42	0.001	−0.47	0.01
ART	−0.42	0.0001	−0.45	0.01
CFFF	0.18	0.01	0.16	0.03

*r* correlation coefficient

In the process of assessing the multicollinearity, the variance inflation factor (VIF) was estimated to find the correlation between the predictor variables and the strength of that correlation. The findings indicated that multicollinearity was not a concern (CFFF, VIF = 1.0; ART, VIF = 1.01, VRT, VIF = 1.07, Gender, VIF = 1.04, BMI, VIF = 1.001). Multiple regression analysis showed that only RTs (for ART, β = − 0.05, t = − 6.75, *p <* 0.001; for VRT, β = − 0.01, t = − 2.3, *p <* 0.02). and gender (β = − 6.84, t = − 5.12, *p <* 0.001) were the significant predictors for academic performance among the students. In contrast, CFFF (β = 0.13, t = 0.11, *p* = 0.27) and BMI (β = − 0.15, t = − 1.09, *p =* 0.27) were not significant predictors in the model for the academic performance (refer to Table 7). The adjusted R^2^ value was 0.11; therefore, 11% of the variation in academic performance would be explained by the model containing RTs and gender.

**Table 7:** Regression analysis of how various factors impact academic performance

Predictor variables	Estimate (β)	Standard error	t value	*P* value
Intercept	79.89	5.33	14.97	0.001
CFFF	0.13	0.11	1.09	0.27
ART	−0.05	0.008	−6.75	0.001
VRT	−0.01	0.007	−2.3	0.02
Gender	−6.84	1.33	−5.12	0.001
BMI	−0.15	0.13	−1.09	0.27

## Discussion

The academic performance of students is affected by diverse factors, and the identification of these is essential for improving the outcome of scholastic achievement and future occupational outcomes. This study aimed to assess whether certain cognitive functions have a role in the academic performance of university students at the Faculty of Medicine and the Faculty of Dentistry. We evaluated the cognitive functions of students by using the ART, VRT and CFFF tests. The results from our study indicate that faster RTs were one of the predictors of better academic performance among the recruited participants. It was observed that female students had faster RTs and that gender was an independent predictor of academic performance.

Faster RTs are indicative of better cognitive functions, including memory and verbal fluency [6], processing speed [34], and intelligence [35–37]. Therefore, students with faster RTs are more likely to have better academic performance, as revealed by other studies. Prabhavathi et al. studied the impact of RT on academic performance among undergraduate medical students [20]. They found that students with faster RTs had higher academic scores, and they attributed it to better attention, concentration, cortical arousal and processing speed. Sharma et al. [28] found similar results in the case of another cohort of medical students, although the correlation was small and not statistically significant. Studies on adolescents also indicate a correlation between cognitive tests and academic performance [18, 38]. However, academic performance may be affected by other factors, and RT is only a single contributor to the various cognitive functions [39, 40]. Academic stress is shown to influence academic scores to a greater degree in the case of female students than in the case of male students [41, 42]. Moreover, other non-cognitive factors, such as gender, age, BMI, attendance percentage, social factors and general health play a role in academic performance [5, 43].

Similar to other studies, VRT was slower in the case of underweight and overweight students, and ARTs were similar [44, 45]. The reasons for this are still unclear, and other confounding factors may be relevant. The arm to height ratio can negatively affect RTs but not in a linear fashion [46]. The BMI has a positive correlation with fat percentage [47, 48] and a complex correlation with muscle mass, handgrip strength and endurance [49]. All these factors can affect RTs individually, with a generally positive correlation between fat percentage and VRT and a negative correlation between muscle function indices and RTs [44, 50–52].

The female students included in our study had a significant RT when compared to the male students, contrary to the findings of the wider literature [18, 20, 52–57]. The different conduction velocities of central neurons, analytical pathways complexity, acetylcholine synthesis and hormonal effects on neural transmission [53] are thought to result in females having faster decision times [58] and faster auditory latencies [59] but slower RTs. In contrast, although the muscle contraction times are similar between genders [60], female students have weaker motor responses [53], and this may further explain the differences in the RTs.

More recent reports show there are no differences in the RTs between genders [34, 61, 62], and this could represent the effect of factors such as the increasing trend of exercise and training among female students, as it is related to faster RTs [52]. RTs in females are also subject to timing with respect to the menstruation cycle [63]. RT can be influenced by the arm span to height ratios, which are different between males and females [46]. Our study did not collect data on exercise or menstruation, and this could be the focus of future research to explain the contrasting results.

Female students had higher academic scores, similar to other studies that have shown an advantage of female students over the male in terms of academic achievement, especially in the health sciences [64, 65], although sometimes, the difference is not statistically significant [20]. A recent meta-analysis confirmed that the wider literature reports similar findings [66], and yet, our regression analysis failed to show gender as a predictor of academic scores. As other authors argue, genders are more alike than different [67], and socioeconomic status, stereotype manipulation and school-related factors may explain most of the academic differences [68].

The data obtained from the literature on the correlation between CFFF and academic performance are scarce and indirect. Several studies have reported that the frequency of CFFF affects several visual processing skills, such as reading, visual attention and alertness [69]. The visual processing speed is essential for scholastic achievement and academic performance because it is directly correlated to reading ability, decision making and cerebral arousability [16, 17, 70]. Corr et al. reported that CFFF was positively correlated with procedural learning [71], and Mewborn et al. also reported that CFFF correlated significantly with executive functions in young adults [11]. Executive functions include working memory, impulse control, cognitive flexibility in generating different solutions to a problem and planning towards achieving an objective that are considered to predict academic performance, at least in the case of primary school children [72]. Caultela and Barlow reported in their study on 40 Boston College undergraduates that there was a significant correlation between CFFF and intelligence measured by the Otis Quick Scoring Intelligence test and the College Board tests for Verbal and Mathematical ability [73], results that have been suggested by other studies [74]. Intelligence is a strong predictor of academic achievement [72], with prior academic achievement also playing a significant role in the pathway between intelligence and final academic achievement [75]. However, CFFF performance is not a predictor of global cognition [11], and our study only revealed a very weak correlation between CFFF and academic scores. The correlation between CFFF and the factors known to affect academic achievement may not be as strong as predicted; more contemporary studies are needed to retest these assertions.

The message from this study is that having data on basal the cognition levels of students is always beneficial, and based on it, cognitive skills can be trained and enhanced [76, 77]. Teachers are recommended to employ cognitive learning strategies that might enhance a learner’s capability to process knowledge more deeply and help them to eventually transfer the knowledge gained and apply it to newer circumstances [78].

Some of the techniques include the following:

1. Spaced practice: “*Creating a study schedule that spreads study activities repeated over a period of time*” [79].

2. Interleaving: “Switching between topics while studying” [79].

3. Elaboration: “Asking and explaining why and how things work” [79].

4. Retrieval practice: “Bringing learned information to mind from long-term memory” [79].

5. Reflection training [80] and Reflection [81, 82].

6. Mindfulness learning [83].

Globally, all medical schools are marching towards a competency-based curriculum where ‘reflection’, ‘case-based discussions’, ‘journal clubs’ and ‘self-directed learning’ are becoming an essential aspect of the effort to improve cognition [81].

One of the main strengths of our study is that it evaluated more than one cognitive domain in correlation with academic performance. Other strong points of this study include large sample size and homogeneity of the study participants in terms of a similar age group, comparable socioeconomic background, school curriculum and qualification through a standard eligibility examination conducted by the Government of India. Other confounders such as gender and BMI were addressed through statistical analysis. The main limitation of the study is that the cognitive tests were not conducted on the same day as the internal assessment exams. However, cognitive tests (including the RT tests) have been reported to have high test re-test reliability [84, 85]. Additionally, the narrow standard deviation values for academic performance indicate that the scores lie within a narrow area, and therefore, it is more difficult to identify the correlations. This was a cross-sectional study, and therefore, we could not establish a causal relationship. The study participants belonged to a homogenous group; hence, other confounding variables (e.g., age, socioeconomic status and educational background) could not be studied. There was no longitudinal follow-up of the study participants.

## Conclusion

Faster VRTs and ARTs are correlated with better academic performance among undergraduate students, and the correlation is independent of other variables such as gender or BMI. The CFFF was practically not correlated. This indicates that attention, concentration, cortical arousal and processing speed may be more important for learning. This study highlights the importance of RT in academic performance. RT can be promoted by following a healthy lifestyle.

## Supplementary Information

**Additional file 1: Supplementary Figure 1** - Block diagram of the Auditory and Visual reaction time measuring device using Audacity® software. **Supplementary Fig. 2** - PC 1000 HZ Reaction timer device. **Supplementary Fig. 3:** Graphical flow chart for Reaction time estimation in Audacity® software with PC 1000 Hz reaction timer. **Supplementary figure: 4** - CFFF measuring portable device. **Supplementary figure: 5** – NETHRA- CFFF device Control software for execution of CFFF test.

## Data Availability

The datasets used and/or analysed during the current study are available from the corresponding author on reasonable request.

## Abbreviations

ART: Auditory reaction time
BMI: Body Mass Index
CFFF: Critical Flicker Fusion Frequency
IQR: Interquartile range
RT: Reaction time
SD: Standard deviation
VRT: Visual reaction time
