# Supplementary material for: How does cognitive function measured by the reaction time and critical flicker fusion frequency correlate with the academic performance of students?
Source: BMC Med Educ. 2020 Dec 14;20:507. doi: 10.1186/s12909-020-02416-7 (PMC7734712; doi:10.1186/s12909-020-02416-7)
Supplement: Supplementary file 1 — Additional file 1: Supplementary Figure 1 - Block diagram of the Auditory and Visual reaction time measuring device using Audacity® software. Supplementary Fig. 2 - PC 1000 HZ Reaction timer device. Supplementary Fig. 3: Graphical flow chart for Reaction time estimation in Audacity® software with PC 1000 Hz reaction timer. Supplementary figure: 4 - CFFF measuring portable device. Supplementary figure: 5 – NETHRA- CFFF device Control software for execution of CFFF test. [file 12909_2020_2416_MOESM1_ESM.docx]

**Supplementary figure 1- Block diagram of the Auditory and Visual reaction time measuring device using Audacity® softwar**e

Computers microphone input for recording the signal

Subject Key switches off the circuit

Examiner key switches on the circuit

DC 5V supply

1KHz square wave oscillator

Flip flop circuit - for switching the oscillator on and off.

Output connected to LED / head phone

**Methodology of measuring reaction time:**

Visual Reaction Time and Auditory Reaction Time are assessed using PC 1000 Hertzs reaction timer. PC 1000 Hertzs reaction timer is a 1000 hertz square wave oscillator which has a soft key for start and stop function. It has two components (E &S) connected to each other. First component (E) has a start button which will be out of the view from the subjects and it is controlled by the examiner only. Second component (S) has a stop button which will be operated by the subjects. Also, it has a small red LED light for visual stimulus and head phone (1000 hertz’s tone) which receives auditory stimulus respectively. Red light is selected for the experiment as it persists for a long time in retina. Component E and component S are in turn connected to a personal computer which has audacity sound software installed in it. Audacity software records the reaction time in 0.001 sec accuracy in wave format.

***
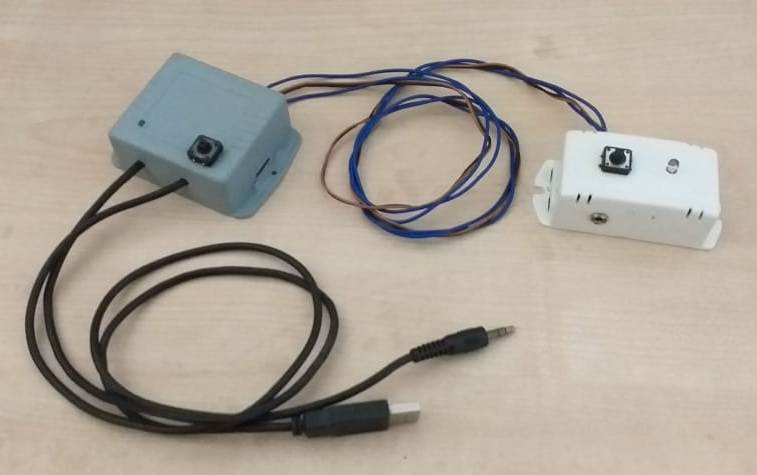
***

***Component E***

***Component S***

**Supplementary figure. 2 - PC 1000 HZ Reaction timer device**


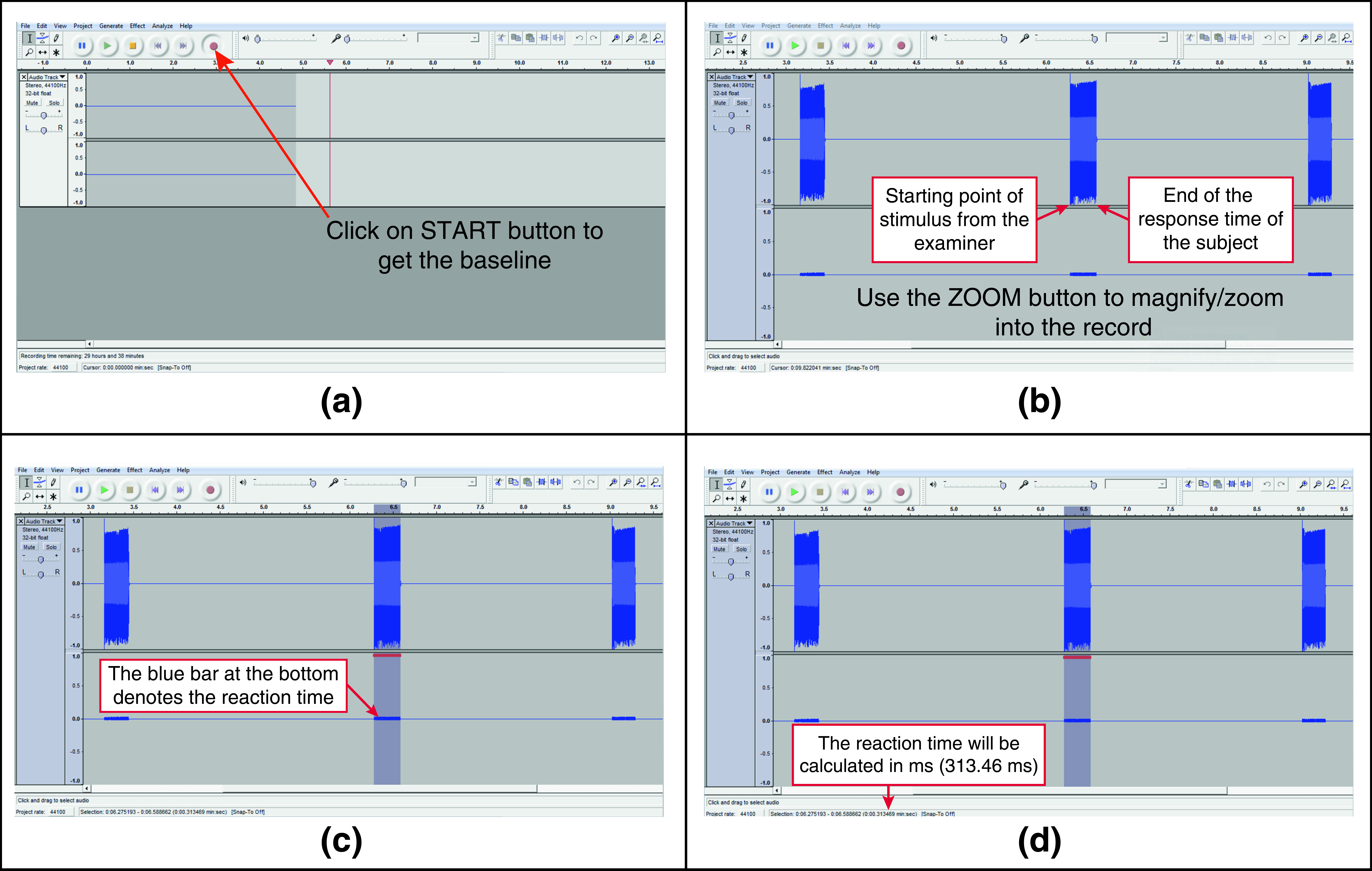


**Supplementary figure 3: Graphical flow chart for Reaction time estimation in Audacity® software with PC 1000 Hz reaction timer**

**Measurement of Critical Flicker Fusion Frequency (CFFF)**

CFFF was measured with a standard electronic module. In this electronic module, the system presents a series of red light stimuli with the help of NETHRA CFF device Control software at different frequencies ranging from 12 – 120 Hz. NETHRA CFF device Control software is a lightweight and portable application that turns a PC into an Audio Oscillator. NETHRA CFF device Control software uses the sound card in the PC to produce sine waves that are mathematically correct and accurate. The red light was surrounded by a white background. A short practice was given to all the subjects following which each participant was seated in front of the electronic module around 80 cms from the stimulus in a semi-dark room provided with a single 40-w bulb fixed behind the participant. After ensuring all the pre-requisites, the frequency of flicker was slowly increased from minimum threshold of 12 Hz till the participants reported that they perceived the successively presented light stimuli to be ‘fused’ or ‘steady’ or ‘constant’ as shown in Fig 13. The resultant data was obtained automatically from NETHRA CFF device Control software. The mean value of three descending measures from a high frequency to a low frequency of flicker in which the students reported when the light stimulus began to flicker, and three ascending measures from a low frequency to a high frequency in which the students reported when the flicker stopped was taken as a measure of CFFF.


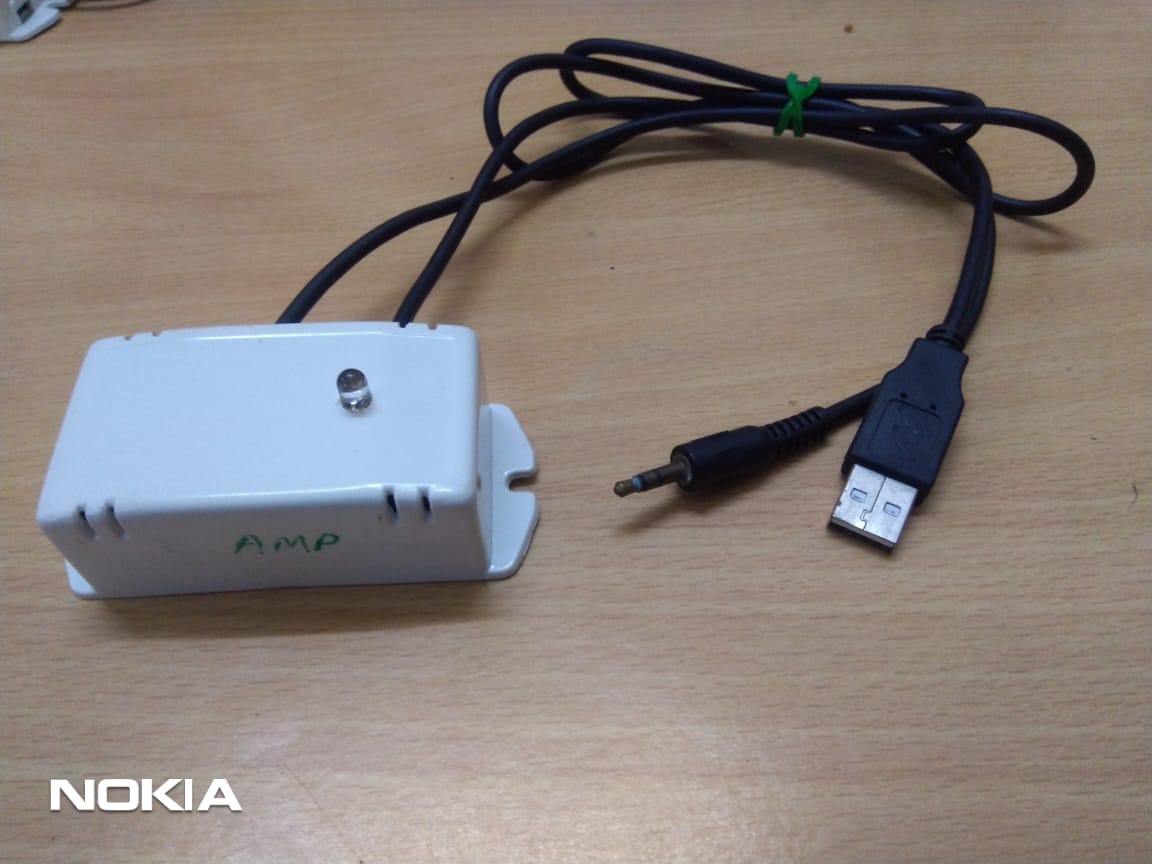


**Supplementary figure: 4 - CFFF measuring instrument Portable device**


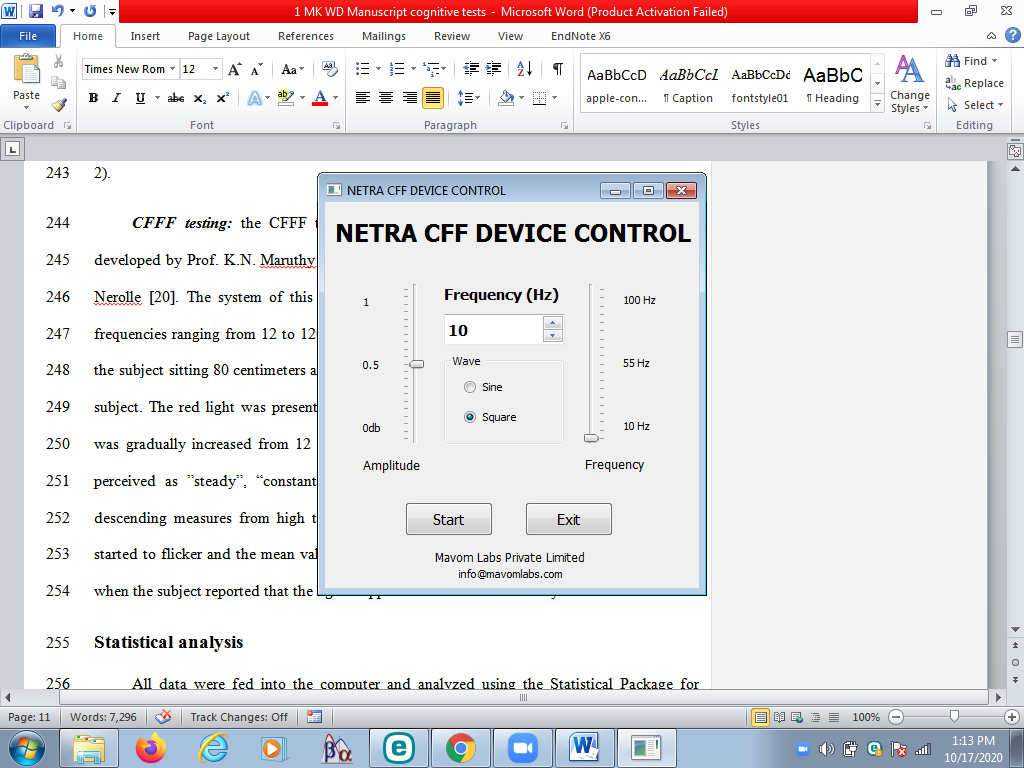


**Supplementary figure: 5 – NETHRA CFF device Control software for Execution of CFFF test**

**Reference:**

1. Kumar AP, Mahesh Kumar K, Padmavathi R, Maruthy KN, Sundareswaran. Validation of PC 1000 Hz reaction timer with biopac® MP 36 for recording simple reaction time. Indian Journal of Physiology and Pharmacology. 2019;63:138–44.

2. Rao PS, Yuvaraj S, Kumari TL, Maruti KN, Sasikala P, Kumar SS, Pal R, Reddy VV, Gorantla R, Agrawal A. Cognition, autonomic function, and intellectual outcomes of the paramedical health-care personnel in the hospital settings. Journal of Education and Health Promotion. 2020;9.

3. Kumar CK, Maruthy KN, Sasikala P, Gurja JP, Kumar AV, Kareem SK. Impact of chronic alcoholism on temporal cognition and coordination of motor activity. International Journal of Physiology. 2018;6(4):124-7.

4. Endukuru CK, Maruthy KN, Deepthi TS. A study of critical flickering Fusion frequency rate in media players. International Journal of Physiology. 2016;4(1):144-8.
